# Two-dimensional heart-cut LC-LC improves accuracy of exact-matching double isotope dilution mass spectrometry measurements of aflatoxin B_1_ in cereal-based baby food, maize, and maize-based feed

**DOI:** 10.1007/s00216-014-8003-5

**Published:** 2014-07-12

**Authors:** Andreas Breidbach, Franz Ulberth

**Affiliations:** European Commission, Joint Research Center, Institute for Reference Materials and Measurements, 2440 Geel, Belgium

**Keywords:** Two-dimensional liquid chromatography, Isotope dilution mass spectrometry, Proficiency testing, Certified reference materials

## Abstract

Aflatoxins, mycotoxins of fungi of the *Aspergillus* sp., pose a risk to consumer health and are, therefore, regulated by more than 100 countries. To facilitate method development and validation as well as assessment of measurement capabilities, availability of certified reference materials and proficiency testing schemes is important. For these purposes, highly accurate determinations of the aflatoxin content in the materials used are necessary. We describe here the use of two-dimensional heart-cut LC-LC in combination with exact-matching double isotope dilution mass spectrometry to determine the content of aflatoxin B_1_ in three materials used in a proficiency testing scheme. The serious reduction in ionization suppression afforded by the two-dimensional heart-cut LC-LC had a positive effect on the precision of the measured isotope ratios of the exact-matching double isotope dilution mass spectrometry. This is evidenced by the expanded measurement uncertainty (*k* = 2) of 0.017 μg/kg or 8.9 % relative to a mass fraction of aflatoxin B_1_ in a cereal-based baby food of 0.197 μg/kg. This value is in perfect agreement with the consensus value of this material from a proficiency test (PT) scheme for National Reference Laboratories executed by the European Reference Laboratory for Mycotoxins. The effort necessary to perform the described methodology precludes its frequent use but for specific applications we see it as a valuable tool.

## Introduction

The occurrence in food and feed of aflatoxins, mycotoxins produced by fungi of the *Aspergillus* sp., poses a risk to consumer health. Currently, more than 100 countries, covering 85 % of the global population, have set regulatory limits for mycotoxins in the food chain [1], and aflatoxins are still among the top ten notifications reported in the European Union Rapid Alert System for Food and Feed [2].

A multitude of methods of analysis exist for the determination of aflatoxins in food/feed and recent developments have been summarized by Shephard et al. [3]. Standardized methods of analysis have been made available by AOAC International [4] (994.08 & 999.07) and the European Committee for Standardization [5] to support harmonized implementation of legislative limits. Those widely applied techniques are based on cleanup by either multifunctional adsorption columns or by immunoaffinity columns (IAC) followed by HPLC with fluorescence detection.

With the advent of LC-MS in mycotoxin analysis, methods were developed using the advantages of stable isotope dilution. Rychlik and Asam [6] reviewed their utility for the determination of mycotoxins. Several LC-MS assays for aflatoxins have been described [7, 8], among them also one for multiple mycotoxins including aflatoxins [9].

Isotope dilution mass spectrometry (IDMS) offers the possibility to obtain exceptional accuracy if an appropriate isotopologue of the analyte, the “spike,” is available and precisely characterized in terms of purity, isotopic pattern, and concentration. Its utility became apparent in the 1950s in the field of elemental analysis and in the 1970s for organic analytes [10].

In 1994, Henrion [11] developed the concept of exact-matching double isotope dilution mass spectrometry (EMD-IDMS). In brief, the material to be analyzed is fortified with the spike to generate the sample blend (“normal IDMS”). The same amount of spike is mixed with an amount of reference material of the native analyte to generate the calibration blend (“reverse IDMS”). Since normal and reverse IDMS are used in combination, we talk about double IDMS. The amount of reference material is such that the isotope ratio in sample and calibration blends “exactly” match in this double IDMS approach. “Exactly” in this context means the ratio of the isotope ratios becomes indistinguishable of 1 and all instrument related biases become negligible. For EMD-IDMS purity, isotopic pattern and concentration of the spike are of only secondary interest.

EMD-IDMS has the potential to be a primary ratio method if certain prerequisites are met [12]. As primary ratio method, it provides full traceability with shortest possible connection to SI units. This is of particular interest for the production of certified reference materials [13] or for assigning a value [14] to materials used in a proficiency testing scheme [15–17].

The high-accuracy of EMD-IDMS is afforded by effectively eliminating bias in the instrumental analysis step and reducing uncertainty through measuring the two blends multiple times in direct succession. The price of this accuracy is a multiplication of the analysis time. Therefore, one aim of the separation and detection part of an EMD-IDMS method of analysis is to be as fast as possible. At the same time, signal intensity also has a significant influence on precision. To that end, it is advantageous to load as much sample as possible onto the separation instrument.

Fast analysis time and high sample loads may have a detrimental effect on signal intensity in LC-MS due to suppression of ionization of the analyte in the atmospheric pressure ionization source [18, 19]. One way to address these matrix effects is certainly IDMS, since the spike will undergo the same effects in the ionization source as the analyte and, therefore, the isotope ratio is unaffected up to a certain degree [20]. Yet, increase of chromatographic resolution is a preferable way to reduce matrix effects because, in the case of ion suppression, signal intensity is improved [21], which will also positively affect precision of the measured isotope ratio.

Improving chromatographic resolution *R*_*s*_ between two peaks can be accomplished by increasing plate number *N*, retention *k*, and/or selectivity *α* according to Eq. 1 [22] as follows:

1 $$ {R}_s=\frac{\sqrt{N}\left(\alpha -1\right) k}{4\left(1+ k\right)} $$

Increasing the plate number *N* can be done in two ways: either by choosing longer columns which results in longer analysis time or by choosing smaller particle sizes which might result in a loss of sample loading capacity, if because of pressure limitations, a shorter column size is chosen. An increase of retention *k* also comes at the cost of longer analysis time.

By tailoring selectivity, resolution can be increased without the disadvantages of increasing analysis time or decreasing sample loading capacity. This can be done by combining different stationary phases in-line in a one-dimensional system [23] or with a two-dimensional set-up with a switching valve [24].

The Institute of Reference Materials and Measurements (IRMM) of the Joint Research Center of the European Commission is a major provider of certified reference materials (CRM) and of proficiency testing (PT) schemes. The availability of relevant reference materials greatly facilitates the proper validation of methods of analysis, and the availability of relevant PT schemes facilitates an assessment of their application. This enables the achievement of reliable compliance testing of food/feed commodities which is of paramount interest to ensure their unrestricted global trade.

In this paper, we report the development of a two-dimensional heart-cut LC-LC approach to overcome matrix effects involved in the analysis of aflatoxin B_1_ (AFB_1_) in food and feed, and exact-matching double isotope dilution mass spectrometry to obtain highly accurate mass fractions for materials used in PTs executed by the European Reference Laboratory (EU-RL) for Mycotoxins at IRMM. We developed a measurement procedure for the high accuracy determination of AFB_1_ and established an uncertainty budget that is fully compliant with the principles laid out in the Guide to the Expression of Uncertainty in Measurement (GUM) [25].

## Experimental

### Chemicals and materials

All chemicals were purchased from either Sigma-Aldrich or VWR and where of at least analytical grade. For the mobile-phase, LC-MS CHROMASOLV (Fluka, Sigma-Aldrich) grade water and acetonitrile (ACN) were used. Deionized water was generated by a Milli-Q system (Millipore, Belgium). Formic acid ∼98 % (FA), and ammonium formate (NH_4_FA), as LC-MS grade mobile-phase additives, were purchased from Fluka. An equimolar mix of FA and NH_4_FA of pH 3.7 was prepared as follows: 4.6 g FA and 6.3 g NH_4_FA were mixed and diluted with water to 34 mL. This NH_4_FA pH 3.7 solution was used as additive for the buffered mobile phase and was equivalent to 10 % FA (*v*/*v*).

The certified reference material ERM-AC057 (AFB_1_ in acetonitrile) with a certified mass fraction *w* = 3.79 μg/kg and an expanded measurement uncertainty (*k* = 2) of 0.11 μg/kg (the combined uncertainty contained contributors from purity assessment, stability testing, and certification) was obtained from IRMM. The spike, isotopologue ^13^C_17_-AFB_1_ in ACN (*c* = 0.502 μg/mL), was purchased from Romer Labs-Biopure (Tulln, Austria). All subsequent dilutions of AFB_1_ and the spike were prepared gravimetrically in neat ACN.

PT materials investigated were a maize-based feed material, a neat maize material, and a cereal-based baby food material, all naturally contaminated with aflatoxins and used in an EU-RL mycotoxin PT in 2011. All the above PT materials were packaged as ground powders and of each material three test units were selected at random for the investigation. Analyte-free materials matching the PT materials were from the material pool of the EU-RL for mycotoxins. Absence of analyte signal was verified with the method described here.

### Instrumentation

The 2D LC-LC system consisted of an Accela low-pressure gradient solvent delivery unit and an Accela auto liquid sampler (ALS) as LC1 (Thermo Scientific, Belgium). LC2 was a high-pressure gradient system made up of two LC-20AD pumps with a microvolume mixer and a DGU-20A degasser (Shimadzu Benelux, The Netherlands). The MS was a TSQ Quantum Ultra triple-quadrupole mass spectrometer with a HESI 2 ion source (Thermo Scientific, Belgium).

First-dimension separation was afforded by a Supelco Ascentis C18 column (50 × 2.1 mm, 3-μm particle size) with an Ascentis Express C18 guard column (5 × 2.1 mm, 2.7 μm; Sigma-Aldrich, Germany) at isocratic conditions of 38 % B at 200 μL/min and 40 °C. To prevent the build-up of late eluting substances, a 1.5-min step-up to 90 % B after elution of the analyte was included. Mobil phase A was water/FA (999/1, *v*/*v*) and B was ACN/FA (999/1, *v*/*v*).

A Supelco Ascentis phenyl column (50 × 2.1 mm, 3 μm) at isocratic conditions of 53 % B at 200 μL/min and room temperature was used for the second-dimension separation. Here, a step-up to 100 % B for 1.5 min was also included in the gradient. Second-dimension mobile phase A was water/NH_4_FA pH 3.7 (999/1, *v*/*v*) and B ACN/water/NH_4_FA pH 3.7 (900/99/1, *v*/*v*/*v*). The addition of NH_4_FA led to the suppression of [AFB_1_ + Na]^+^ and increase in [AFB_1_ + H]^+^.

The integrated six-port, two-position divert valve of the TSQ Quantum Ultra was used for the transfer of the heart-cut of the first-dimension separation to the second-dimension column. To achieve this, a 100-μL loop was used to trap the analyte eluting from the first-dimension column. During preliminary tests, the switching time was determined by connecting the outlet of the loop directly to the MS. The retention time of the front of the analyte peak minus the delay caused by the internal volume of the ESI probe and the tubing is the run time at which the analyte peak fills the loop. After switching the content of the loop was loaded in reverse onto the phenyl column which was installed between the valve and the ion source.

For 1D separations, the Shimadzu solvent delivery system was connected to the Accela ALS and a Supelco Ascentis Express C18 column (75 × 2.1 mm, 2.7 μm) with an Ascentis Express C18 guard column (5 × 2.1 mm, 2.7 μm). Mobile phase A and B were identical with the second-dimension conditions above. Separation was performed isocratically at 35 % B, 300 μL/min, and 40 °C.

The MS ion source settings are listed in Table 1. The MS analyzer was used in selected reaction monitoring (SRM) mode with argon as collision gas at 0.2 Pa (1.5 mTorr) and the monitored ions are listed in Table 2. Scan cycle time was set to 0.7 s for the seven transitions measured to record >30 scans per peak.

**Table 1:** Ion source settings of the mass spectrometer; the gas flows are in arbitrary flow units (afu.)

Item	Value
Cap temp	320 °C
Vap temp	250 °C
Spray voltage	2.4 kV
Skimmer	10 V
Ion sweep gas	10 afu.
Aux gas	10 afu.
Sheath gas	30 afu.
Tube lens offset	110 V

**Table 2:** Ions monitored by MS during SRM: the protonated species [M+H]^+^ was selected as precursor at unit resolution

Analyte	Precursor (*m*/*z*)	Product (*m*/*z*)	Collision energy (V)
AFB1	313.1	241.0	37
AFB1	313.1	270.0	29
AFB1	313.1	285.0	23
^13^C_17_-AFB1	330.1	227.0	29
^13^C_17_-AFB1	330.1	284.0	33
^13^C_17_-AFB1	330.1	301.1	23
^13^C_17_-AFB1	330.1	314.1	25

### Blend preparation

To minimize potential within-unit inhomogeneities, the entirety of each test unit (ca. 30 g) of each material was additionally comminuted/homogenized for 15 min with a Mortar Grinder with a hard porcelain grinding set (Retsch, Haan, Germany).

The sample blend (SB) consisted of 2 g test material weighed into a 50-mL conical screw-cap polypropylene centrifuge tube (VWR, Belgium) to which 4 mL of water was added. After the material was fully suspended by vortex mixing, the spike was weighed in. The amount of spike was chosen such that the observed isotope ratio in the SB (*R*_*B*_^′^) of the total ion current (TIC) of analyte ion over spike ion would be near unity.

Calibration blends (CB) consisted of 2 g of a matched analyte-free material. After suspending in 4 mL of water, the same amount of spike as in the SB was added. Then, AFB_1_ was weighed in such that the observed isotope ratio in the CB (*R*_*Bc*_^′^) would also be near unity. All weighing was performed with an analytical balance of readability *d* = 0.01 mg (Sartorius ME235S, Belgium) and weights were recorded with full precision. The balance is recertified annually by the manufacturer and checked daily with a 1-g weight of Class E2 with full traceability to the SI unit.

From each of the three units of the baby food and maize test materials, two SBs were prepared for a total of six SBs per material. Of the feed test material, one unit was used up for preliminary tests and of the remaining two units, three SBs each were prepared for a total of six SBs. One matching CB was prepared per test unit, .i.e., three CBs for baby food, three CBs for maize, and two CBs for feed.

### Preparation of injection solutions and measurements

For extraction, 16 mL ACN were added to the blends. For the feed and maize material, this was done in a single addition; while for the baby food, it was done in four portions with intermediate vortex mixing to prevent the sudden precipitation of the milk protein and the resulting loss of analyte in the precipitate. The blends were then agitated on an orbital shaker (KS 260 control, IKA-Werke, Germany) for 30 min and centrifuged (Centrifuge 5810R, Eppendorf, Germany) at 3,200×*g* for 10 min.

Of the clear supernatant, 4 mL for the baby food and 2 mL for the maize were transferred into silanized glass vials (Supelco 45 × 15 mm, Sigma-Aldrich). After evaporation to dryness under a stream of N_2_ at 70 °C, the dry residues were reconstituted with 120 μL of ACN, vortex mixed, and then diluted with additional 280 μL of water. For the feed, because of the higher contamination, 300 μL of clear supernatant were diluted by addition of 500 μL of water.

Of the reconstituted and diluted solutions, 20 μL were injected (“no waste mode”) without any further treatment. For the determination of matrix effects, the same procedure as above was performed with the feed material but the spike was added after the extraction into an aliquot of clear supernatant.

The measurement batches began with several blank runs until the instrument was fully equilibrated, especially the ion source temperatures. The next injection was a SB followed by a corresponding CB. This pair was repeated ten times and followed by a blank run again. This sequence of ten SB/CB pairs and a blank run was repeated for every SB prepared. Always, SBs of the same test unit shared the respective CB for that test unit. Isotope ratios in the SBs (*R*_*B*_^′^) and CBs (*R*_*Bc*_^′^) were calculated from the TIC of analyte ion over TIC of spike ion.

### Calculations

Since the following assumptions were met, the simplified version [26] of the model equation (Eq. 2) for double IDMS could be used to calculate the mass fraction *w*_*X*,*i*_ of analyte in the *i*th SB: occurrence of the spike ion signal in the native test materials and in the reference material of the native analyte was negligible; occurrence of the analyte ion signal in the spike material was negligible; “exact matching” was achieved.

2 $$ {w}_{X, i}={w}_Z\frac{m_{Y, i}{m}_{Z, i}}{m_{X, i}{m}_{Y c, i}}{\overline{R}}_i^{\prime } $$

where *w*_*Z*_ = mass fraction of analyte in reference material, *m*_*X*,*i*_ = mass of test material in *i*th SB, *m*_*Y*,*i*_ = mass of spike added to *i*th SB, *m*_*Z*,*i*_ = mass of reference material in *i*th CB, *m*_*Yc*,*i*_ = mass of spike added to *i*th CB, and ${\overline{R}}_i$ = mean of all measurements of *R*_*B*,*ij*_^′^/*R*_*Bc*,*ij*_^′^ for the *i*th SB/CB pair with *R*_*B*,*ij*_^′^ = observed isotope ratio of the *j*th measurement of the *i*th SB and *R*_*Bc*,*ij*_^′^ = observed isotope ratio of the *j*th measurement of the *i*th CB.

The combined uncertainty of *w*_*X*,*i*_ can then be expressed by Eq. 3 [25] as follows:

3 $$ {u}_{c, i}\left({w}_{X, i}\right)={w}_{X, i}\sqrt{{\left(\frac{u\left({w}_Z\right)}{w_Z}\right)}^2+{\left(\frac{u\left({m}_{Y, i}\right)}{m_{Y, i}}\right)}^2+{\left(\frac{u\left({m}_{X, i}\right)}{m_{X, i}}\right)}^2+{\left(\frac{u\left({m}_{Z, i}\right)}{m_{Z, i}}\right)}^2+\left(\frac{u\left({m}_{Y c, i}\right)}{m_{Y c, i}}\right)+{\left(\frac{u\left({\overline{R}}_i^{\prime}\right)}{{\overline{R}}_i^{\prime }}\right)}^2} $$

where *u* denotes the standard uncertainty of the respective term of Eq. 2, e.g., *u*(${\overline{R}}_i$) is the standard error of the mean of the ten measured ratios *R*_*B*,*ij*_^′^/*R*_*Bc*,*ij*_^′^ in the *i*th SB/CB pair.

The mass fraction *w*_*T*_ of a test material is then calculated by Eq. 4 as follows:

4 $$ {w}_T={\overline{w}}_X{F}_X $$

where ${\overline{w}}_X$ = mean of all six *w*_*X*,*i*_ of one test material and *F*_*X*_ = a factor of unity representing the mean of the relative combined uncertainties of *w*_*X*,*i*_ of one test material. The combined uncertainty of *w*_*T*_ is then expressed by Eq. 5 as follows:

5 $$ {u}_c\left({w}_T\right)={w}_T\sqrt{{\left(\frac{u\left({\overline{w}}_X\right)}{{\overline{w}}_X}\right)}^2+{\left(\frac{u\left({F}_X\right)}{F_X}\right)}^2} $$

where *u*(${\overline{w}}_X$) = the standard error of the mean of ${\overline{w}}_X$ and *u*(*F*_*X*_) = the mean of all *u*_*c*,*i*_(*w*_*X*,*i*_) / *w*_*X*,*i*_ per test material.

All calculations were performed with R, a language and environment for statistical computing [27].

## Results and discussion

### Method development

The method aimed at minimizing manual sample manipulations and possible sample losses through off-line cleanup, thus maximizing precision. Also, short instrument cycle times are of importance to facilitate the large number of runs necessary for EMD-IDMS. Therefore, existing mycotoxin methods of analysis using IAC or Mycosep® cleanup ([4, 5]) were not considered for this investigation. An on-line IAC/HPLC-FLD method for aflatoxin analysis [28], published around the time this study was performed, would have met the minimum manual handling and on-line cleanup requirements but required an excessive run cycle time.

**Fig. 1:**
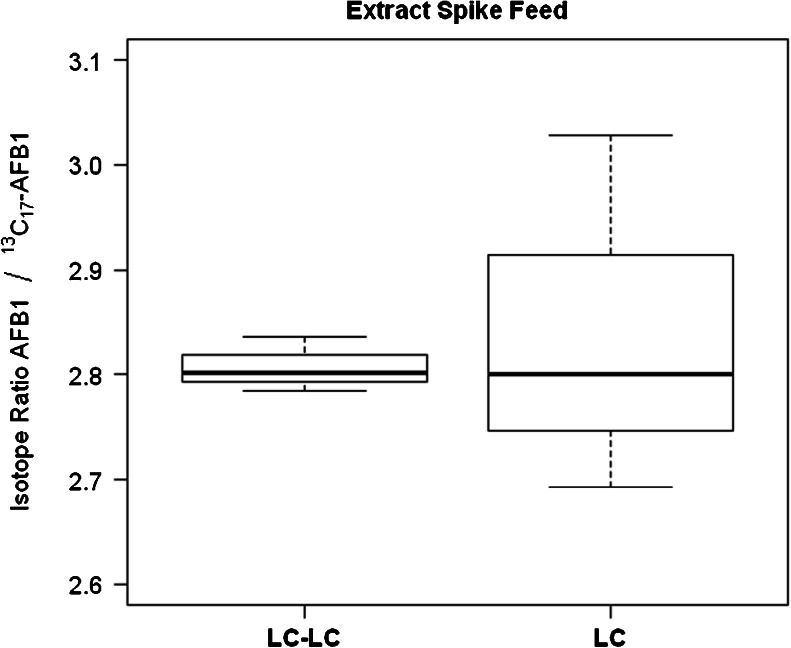
Box and Whisker plot of isotope ratio of analyte ion peak area over spike ion peak area for the feed material spiked volumetrically after extraction

Based on previous experience and published data [29], ACN/water (80/20, *v*/*v*) was chosen as extraction solvent since its extraction yield in cereals is sufficiently high. As fast cycle time was considered to be important, isocratic separation with a very efficient fused-core C18 column was initially selected and MS settings were optimized with a series of designed experiments. With this set-up, injection volumes of crude extract in excess of 5 μL led to peak distortion. It also became apparent that AFB_1_ suffered from significant suppression in the ion source. This is in agreement with what has been reported for aflatoxins in maize [19] and even the use of an analytical column with sub-2 μm particles was unable to alleviate this [9]. While this suppression did not affect the magnitude of the observed isotope ratio (the benefit of IDMS), it did have an impact on the repeatability of the measurements (Fig. 1).

Since heart-cut LC-LC can improve resolution and, thereby, minimize matrix effects, a 50 × 2.1 mm C18 column with 3-μm particles was combined with a 50 × 2.1 mm, 3 μm, phenyl column. This particle size would still deliver a sufficient plate number while having enough sample loading capacity to accept larger injection volumes than the fused-core column. Still at 20 μL per injection, peak shape was acceptable. The combination of the different selectivities α of the two columns obviously provided better separation from matrix constituents and less ion suppression. The higher retention of AFB_1_ on the phenyl column led to focusing of the transferred heart-cut which resulted in a better peak shape.

To limit overall cycle time, complexity, and, therefore, development time, both separation dimensions were run isocratically. The mobile phase conditions were chosen such that the retention factor *k* of AFB_1_ on both columns was between 2 and 3. Beyond this retention, the increase in resolution is outweighed by the increase in analysis time. The C18 column was connected to the six-port, two-position divert valve of the MS. In the “Load” position, the flow of the C18 column went through a 100-μL loop and then to waste. As soon as the AFB_1_ peak was expected to elute from the first-dimension column and was trapped in the loop, the valve was switched to “Inject” by the MS control software. The valve switch time was optimized for maximum signal of AFB_1_. The loop content was then loaded in reverse flow onto the second-dimension column. To keep extra column volumes at its minimum, the phenyl column was mounted between the valve and the ion source.

### Matrix effects

**Fig. 2:**
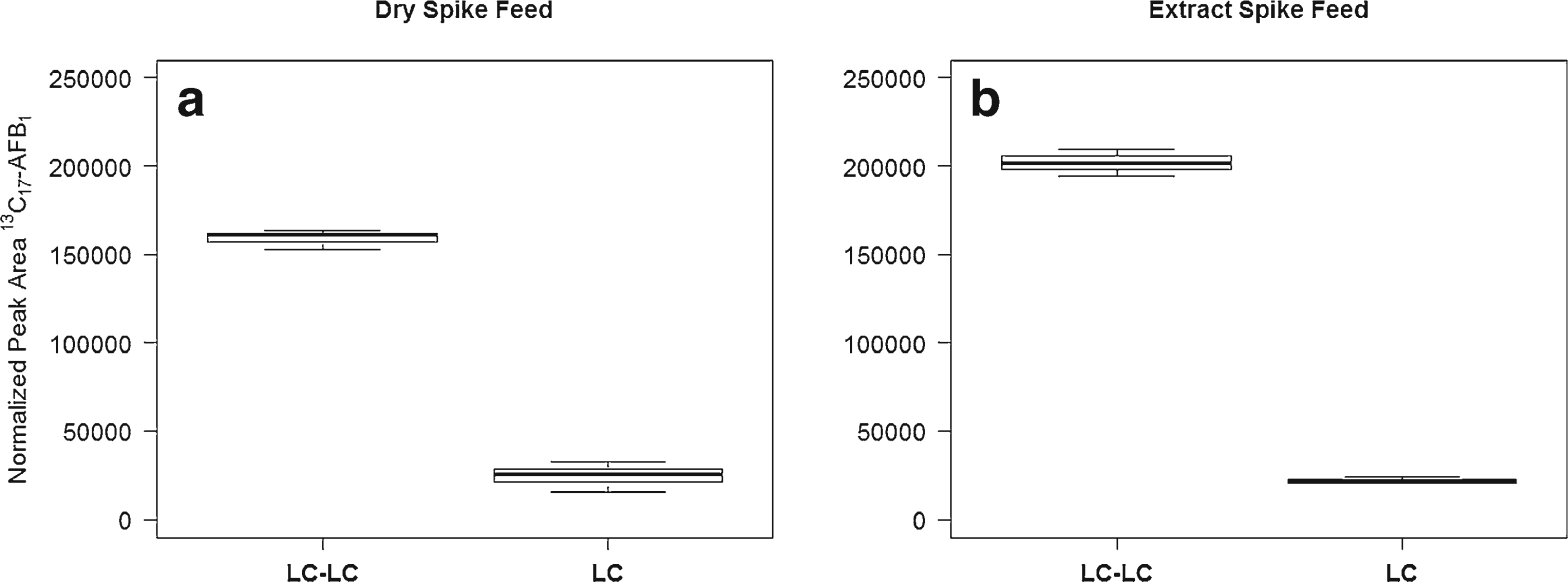
Box and Whisker plots of spike ion peak areas normalized to area per μL injection volume for the feed material spiked volumetrically before extraction (**a**) and the same native material spiked after extraction (**b**); each set represent three repeated injections

The improved chromatographic resolution resulted in significantly less ion suppression (Fig. 2). To restore comparability between the 20 μL injection volume for LC-LC and the 5 μL for LC, peak areas were normalized to area per μL injection volume. The left panel (a) shows the normalized peak areas of the spike ion in the feed material spiked volumetrically once before extraction, the right one (b) shows the same for the feed material spiked after extraction. Both plots show the severe suppression of the signal in 1D-LC. The fact that the LC-LC peak area for the “spike before extraction” is slightly smaller than for the spike added to the crude extract indicates that the extraction efficiency is not 100 % but still acceptable.

**Fig. 3:**
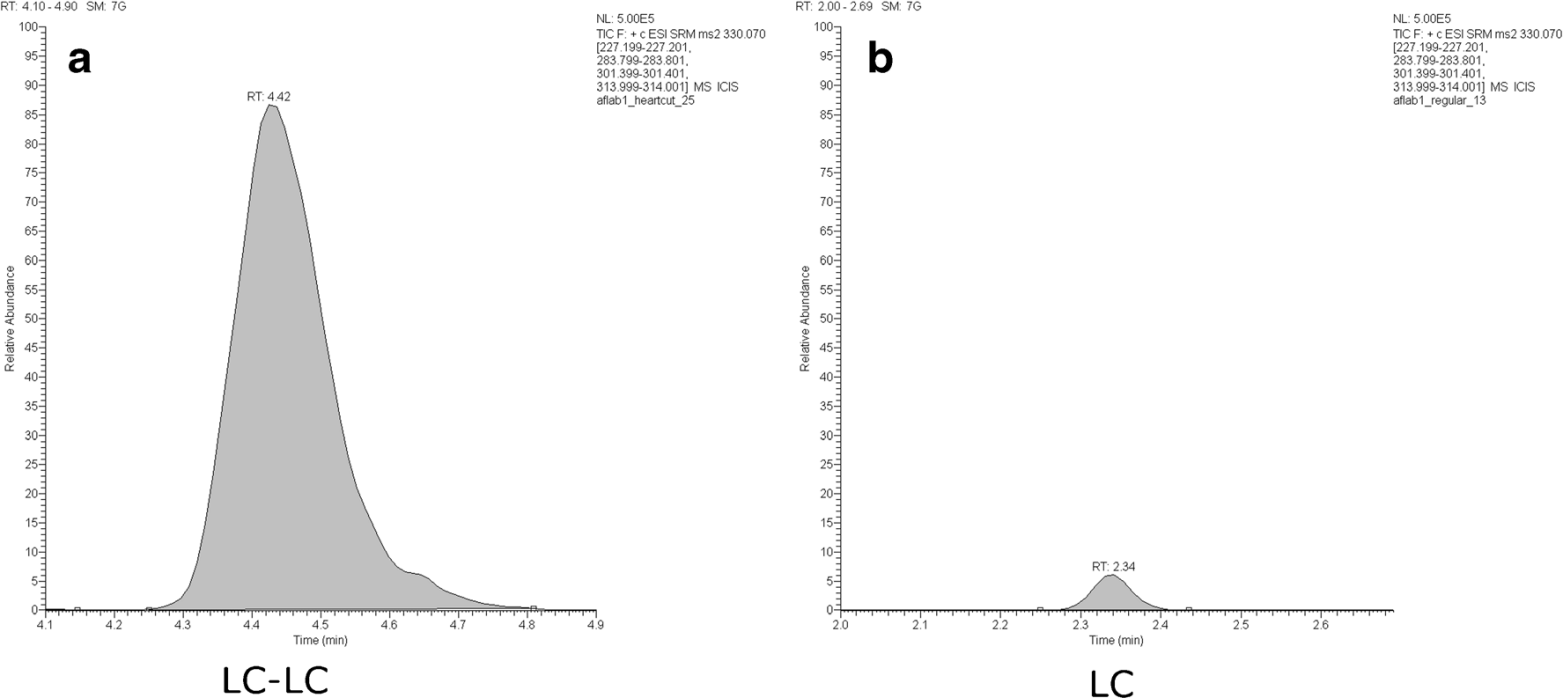
*Left*-*hand panel* (**a**) shows the extracted ion current of the spike ion after LC-LC of an injection of 20 μL of the native feed material extract spiked after extraction, the *right*-*hand panel* (**b**) after LC of 5 μL of the same solution

Figure 3 shows the actual peak size for 2D-LC (a) and 1D-LC (b) in the feed material spiked after extraction. It can easily be recognized that the increase in peak area is much larger than the increase of injection volume by a factor of 4 would suggest. Together with the reduced ion suppression, the peak area in 2D-LC was on average 37 times larger than in 1D-LC.

Figure 1 depicts the observed isotope ratio. The two medians (bold horizontal line) are basically identical for the two approaches showing the potential of IDMS to provide unbiased results even in the presence of severe matrix effects. It is also apparent that the dispersion is much smaller for 2D-LC which we attribute to the much larger signal having a pronounced positive effect on repeatability even though the 2D-LC peak displays a certain asymmetry (Fig. 3a).

### Exact-matching double isotope dilution mass spectrometry

Preliminary mass fractions of AFB_1_ in the three PT materials were known from homogeneity tests. Based on that information, dilutions of the AFB_1_ (ERM-AC057) and the ^13^C_17_-AFB_1_ were prepared such that the amount to be added to the blends could be handled with either a 100 μL or a 500 μL microsyringe. Furthermore, the test materials, the ERM-AC057, and the ^13^C_17_-AFB_1_ were analyzed in their native states to confirm that the assumptions for Eq. 2 were met. The isotope ratios *R*_*X*_ in the test material and *R*_*Z*_ in the reference material were much larger than 1, and the isotope ratio *R*_*Y*_ in the spike was much smaller than 1, thus there was no indication that the simplified double IDMS model equation (Eq. 2) was inappropriate. In organic analysis, these assumptions usually hold true.

**Fig. 4:**
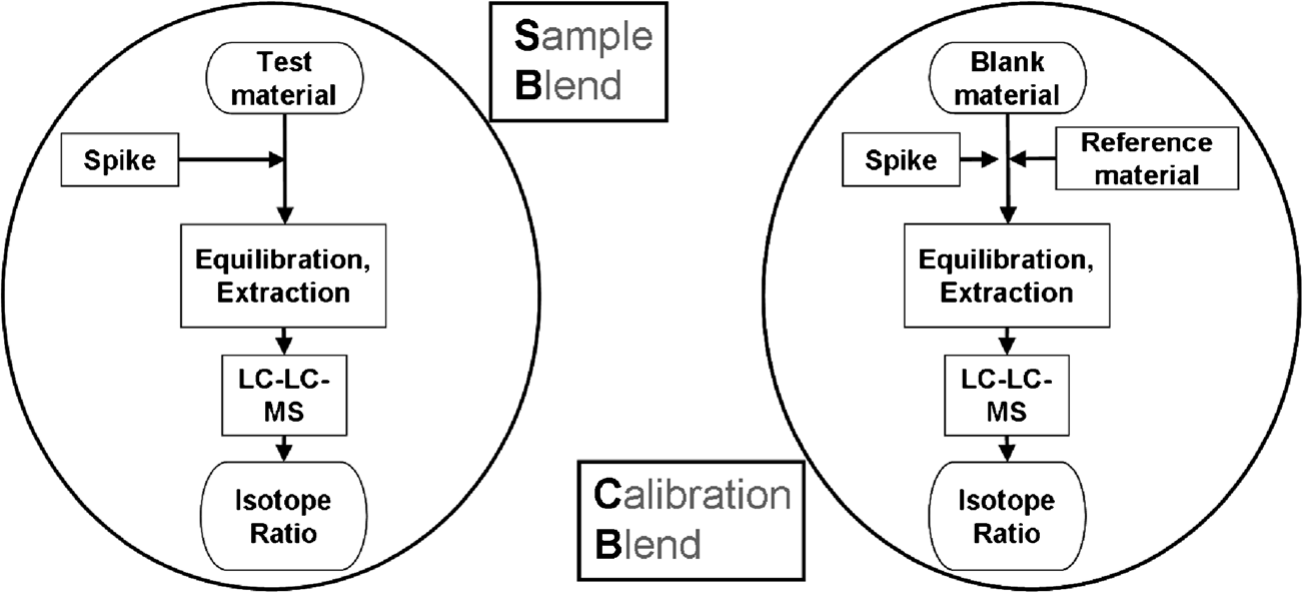
Flow scheme of the EMD-IDMS process

Figure 4 displays the flow of the process of the preparation of sample and calibration blends. To achieve “exact-matching” several iterations of preparations and measurements were necessary, in our case three to four. We started out with the test material and added an amount of spike that would result in a *R*_*B*_^′^ ∼ 1 based on a preliminary estimation of the AFB_1_ mass fraction. At the same time, an analyte-free matched material was fortified with the same amount of spike plus an amount of the reference material to also obtain a *R*_*Bc*_^′^ ∼ 1.

High accuracy with IDMS can only be attained if the native analyte contained in the sample and its added isotopologue reach equilibrium. During the first iteration, the time for reaching equilibrium was investigated. To that end, 2-mL aliquots of the supernatant were withdrawn at 10, 50, 100, and 1,140 min. The raw extract aliquots were centrifuged as described to pellet particulate matter, the clear supernatant evaporated to dryness, reconstituted and injected. No significant correlation between isotope ratio and extraction time could be established indicating equilibration was reached very quickly thus an extraction time of 30 min was chosen.

Based on the observed isotope ratios, the amounts of spike added to both blends, and reference material added to CB were adjusted and a new iteration was performed. That process was repeated until “exact” matching was achieved. Once the right amounts of spike and reference material were known, the six SBs and three (two for feed) CBs per test material were prepared for the final measurement campaign.

**Fig. 5:**
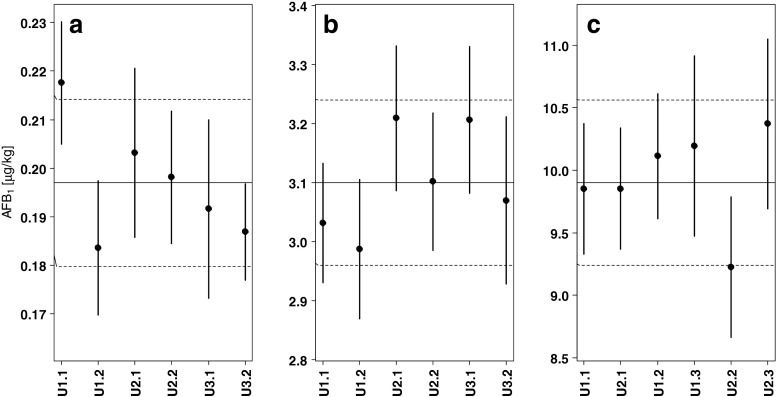
Distributions of the results of the six test units per PT material: **a** baby food, **b** maize, **c** feed; y scale has been adjusted such that the expanded uncertainties of the assigned values have equal width, x labels identify test unit number and repetition (U1.1: unit one, first repetition); *solid circle* result, *vertical lines* expanded uncertainties, *horizontal solid line* assigned value, *horizontal broken lines* expanded uncertainty of assigned value

Table 3 shows that the uncertainty budget for the baby food worked out following the GUM principles. The main contributors to the uncertainties of the individual mass fractions (*u*_*c*,*i*_(*w*_*X*,*i*_)) are the observed isotope ratios and the main contributors to the uncertainty of the total mass fraction (*u*_*c*_(*w*_*T*_)) are the individual *u*_*c*,*i*_(*w*_*X*,*i*_). The contribution of all mass determinations to the overall combined uncertainty is negligible (<1 ‰). Panel a in Fig. 5 depicts the individual mass fractions for the six aliquots of the baby food with their expanded uncertainties and the respective total mass fraction of AFB_1_ in the test material. Panels b and c in Fig. 5 show the same for the maize and the feed material for which the contributions to their respective uncertainty budgets are similar to baby food (data not shown).

**Table 3:** Uncertainty budget for the baby food material

Item	Value	Contribution
*w*_*Z*_	2.091(30) ng/g	*u*(*w*_*Z*_) 25 %
*m*_*Y*,1_	0.23302(07) g	
*m*_*X*,1_	2.00594(01) g	
*m*_*Z*,1_	0.22059(07) g	
*m*_*Yc*,1_	0.23302(07) g	
${\overline{R}}_1$	0.937(23)	*u*(${\overline{R}}_1$) 75 %
*w*_*X*,*i*_	*0.217*(*6*) *ng*/*g* 0.184(7) ng/g 0.203(9) ng/g 0.198(7) ng/g 0.191(9) ng/g 0.187(5) ng/g	
*w*_*T*_	0.197(9) ng/g	*u*(${\overline{w}}_X$) 34 % *u*(*F*_*X*_) 66 %

The first six rows represent the terms of Eq. 2; the row labeled *w*_*X*,*i*_ lists the six individual results (in *italics* the result corresponding to the first six rows); *w*_*T*_ is the result of Eq. 4; the number in parentheses is the numerical value of *u*_*c*_ referred to the corresponding last digits of the quoted result; the last column shows the percent contribution to the respective *u*_*c*_

The expanded (*k* = 2) measurement uncertainty of 8.9 % for the baby food material containing 0.197 ng AFB_1_/g material attests to the exceptional accuracy achievable with EMD-IDMS. That the effort of the described method is well worth it and necessary for assigning a reference value to a material can be seen if the result of the maize material (3.1 ± 0.14 μg/kg, 4.6 %, *k* = 2; see Table 4) is compared to published data of similar contamination. Cervino et al. [7], using deuterated aflatoxin B_2_ for their IDMS assay, reported a relative interassay precision of 12 % for AFB_1_ at a level of 4.2 ng/g (NIST SRM 2387, peanut butter), and Li et al. [8] 7–11 % for AFB_1_ spiked at a level of 4 ng/g to various feed matrices. Varga et al. [9] report for a contamination range of 2–12 ng/g relative standard deviations of 4–6 %. It has to be borne in mind that in all three studies, the advantages of IDMS were not fully exploited (Cervino used d_3_-AFB_2_, Li and Varga added the isotopologue after the extraction) and the cited data refer to estimates of precision and, therefore, present only part of the uncertainty of measurement.

**Table 4:** The robust means of the proficiency test (PT) and the assigned values with their uncertainties determined with this study

Material	PT robust mean (μg/kg)	Assigned value *x*_*a*_ (μg/kg)	Expanded uncertainty U(*x*_*a*_) (μg/kg)	Relative expanded uncertainty (%)	Coverage factor (k)
Baby food	0.20	0. 197	0.017	8.9	2
Maize	2.8	3.1	0.14	4.6	2
Animal feed	8.6	9.9	0.66	6.7	2

The trueness of this reference measurement procedure was verified by comparing the mass fraction for baby food to the robust mean determined from the EURL-Mycotoxin PT 2011 (Table 4) [30]. The more than 60 participating laboratories represented the National Reference Laboratories of all 27 Member States of the European Union (status as of 2011) plus a number of selected Official Food/Feed Control laboratories. For the other two materials, the robust mean of the PT is just short of the expanded uncertainty range around the assigned value. The reason for this could be improper recovery correction. The vast majority of the participating laboratories performed immunoaffinity clean-up. If the recovery determination was performed with a material not sufficiently matching the test material, the actual recovery might be misjudged.

## Conclusions

While isotope dilution mass spectrometry is capable to deliver unbiased results even in the presence of severe matrix effects, the control of these effects through appropriate measures will improve accuracy. We used heart-cut LC-LC as a strategy to increase chromatographic resolution and, by that, reduce ion suppression experienced in the analysis of aflatoxin B_1_ in maize-based feed, neat maize, and cereal-based baby food. The increase in analyte signal afforded by the increased ionization yield and the larger injection volume compared to an analytical column with fused-core particles led to improved precision. This is evidenced by the mass fraction of AFB_1_ in baby food determined to be 0.197 μg/kg with an expanded measurement uncertainty of 0.017 μg/kg or 8.9 %. Given the low contamination level and the simple, straightforward sample preparation, this is remarkable.

The effort necessary to perform EMD-IDMS precludes its frequent use but in particular settings, like assigning a value to a material for its use in a PT or as certified reference material, or in the case of referee analysis to dissolve disputes, it is a valuable tool.
